# A register-based study on associations between pre-stroke physical activity and cognition early after stroke (part of PAPSIGOT)

**DOI:** 10.1038/s41598-022-09520-2

**Published:** 2022-04-06

**Authors:** Malin Reinholdsson, Tamar Abzhandadze, Annie Palstam, Katharina S. Sunnerhagen

**Affiliations:** 1 Departments of Clinical Neuroscience, Institute of Neuroscience and Physiology, Sahlgrenska Academy, University of Gothenburg, Per Dubbsgatan 14, fl. 3, 413 45 Gothenburg, Sweden; 2 Department of Occupational Therapy and Physiotherapy, Sahlgrenska University Hospital, Gothenburg, Sweden; 3 Rehabilitation Medicine, Neurocare, Sahlgrenska University Hospital, Gothenburg, Sweden

**Keywords:** Medical research, Neurology, Risk factors

## Abstract

The objective was to investigate if pre-stroke physical activity is associated with intact cognition early after stroke. The study design was a cross-sectional, register-based study. The study sample included 1111 adults with first stroke (mild or moderate severity) admitted to three Swedish stroke units. The main outcome was cognition. The associations of pre-stroke physical activity, age, sex, smoking, diabetes, atrial fibrillation, previous TIA, statin treatment, hypertension treatment, reperfusion therapies, stroke severity, and education on the outcome cognition were analyzed using binary logistic regression. Physical activity was assessed within 48 h of admittance, and cognition was screened during stroke unit care. The results were: mean age 70 years, 40% women, 61% pre-stroke physically active, and 53% with post-stroke cognitive impairment. Patients with pre-stroke light or moderate physical activity have higher odds for intact cognition compared to inactive: odds ratio (95% confidence interval) 1.32 (0.97-1.80) and 2.04 (1.18-3.53), respectively. In addition to pre-stroke physical activity, people with younger age, a higher level of education, less severe stroke (more mild than moderate), being non-diabetic, and non-smoking have higher odds for intact cognition. In conclusion physical activity before stroke is associated with intact cognition in patients with mild and moderate stroke.

## Introduction

Stroke affects one in four during a lifespan^1^, and even though stroke incidence is stable and mortality rates are decreasing, the number of people living with the consequences of stroke is increasing^2^. Common symptoms of stroke are decreased motor skills and sensory functions, visual loss, aphasia^2^, and cognitive impairment. Cognition includes all processes of thinking used in interaction with others and the environment, in a complex system of varied cognitive functions from visual perception to social cognition^3^. Cognitive impairment after stroke is common but the prevalence differs from 20 to 70% in studies, which can be explained by the use of different diagnostic criteria and assessments^4^. In a Swedish study, the prevalence was 59% after acute stroke^5^, and according to another study 71% of the patients without functional disability after 3 months had cognitive impairment^6^. Risk factors for cognitive impairment after stroke are higher age, lower level of education, and vascular risk factors, and they vary between occupations, countries, and ethnicities^4,7^. Post-stroke cognitive impairment can affect various activities and may thereby cause disability^3^. Cognitive screening early after stroke using the Montreal Cognitive Assessment (MoCA) can predict early dependency in activities of daily living^8^, but is also associated with long-term cognitive outcome, functional disability, activities of daily living, and mortality^9^.

As many as 80% of strokes can be prevented by modifying risk factors^10^, and physical activity can reduce the risk for stroke by 25%^11^. Higher levels of pre-stroke physical activity have been associated with less acute stroke severity, better functional outcomes on the modified Rankin Scale and better performance in activities of daily living^12,13^. Physical activity promotes neuroprotective mechanisms and neuroplasticity, where neurogenesis and angiogenesis interact in regenerative and repair processes, and also reduces inflammation and increases levels of growth factor^12^. The definition of physical activity is any body movement that expends energy^14^. Exercise is a subcategory of physical activity that is planned, structured, and repeated to improve or maintain fitness^14^. Physical activity post-stroke seem to have a small to moderate positive effect on cognition after stroke^15^. Regarding associations between pre-stroke physical activity and cognition, one previous study with 625 participants reported associations of pre-stroke physical activity on faster processing speed but not global cognition^16^. Another study with 694 participants reported physical activity not to be a predictor of cognitive decline after stroke^7^. Related to the topic, a study with 88 participants reported that light physical activity but not physical exercise was associated with a reduction of post-stroke dementia^17^. How pre-stroke physical activity influences cognition after stroke needs to be further investigated. The objective of the present study was to investigate if pre-stroke physical activity is associated with intact cognition early after stroke.

## Methods

### Study design

This is a cross-sectional, non-interventional, register-based study, and a part of the project Physical Activity Pre-Stroke in Gothenburg (PAPSIGOT)^18^. Retrospective data were retrieved from two Swedish stroke registers, the national stroke quality register (Riksstroke)^19^ and a local stroke register (Väststroke). The two registers provide different information about the same patients. The datasets were merged through personal identification numbers into one pseudonymized database. The project was registered at www.researchweb.org, project number 214641. This article was written according to the Strengthening the Reporting of Observational Studies in Epidemiology (STROBE) guidelines^20^.

The study was approved by the Regional Ethical Review Board in Gothenburg, Sweden on May 4, 2016, registration number 346-16, with an amendment approved on September 14, 2018, registration number T807-18. According to the Data Inspection Board in Sweden data from quality registers are exempt from the general rule of patient consent (the Personal Data Act, Swedish law No. SFS 1998:204), since their purpose is to improve the quality of care, treatment, and rehabilitation which is of public interest. Furthermore, the patients were informed that their data were reported to the stroke registers and could be used for research. The patients may opt-out with withdrawal of their data at any time. The data were anonymized thus patients cannot be identified. The research was performed according to the declaration of Helsinki.

### Study sample

The study sample consisted of patients with stroke admitted in the period from November 1, 2014, until August 31, 2018, to the Sahlgrenska University Hospital. The hospital consists of three hospitals, each with a comprehensive stroke unit, and provides emergency and basic care for 700,000 inhabitants in Gothenburg as well as specialized care for 1.7 million inhabitants in the west of Sweden (Västra Götaland). Subjects matching the inclusion criteria were adults (≥ 18 years) with the diagnosis ischemic stroke (I63) or intracerebral hemorrhage (I61) according to the International Classification of Diseases (ICD-10), previously living independently in the community, Swedish speaking, and with assessments for pre-stroke physical activity and post-stroke cognition. Exclusion criteria were acute severe or very severe stroke severity assessed as 15–42 points on the National Institutes of Health Stroke Scale (NIHSS)^21^, and previous stroke.

### Procedures

Trained nurses registered the medical and nursing care variables gathering data from medical charts, while physiotherapists assessed and registered pre-stroke physical activity using the Saltin-Grimby Physical Activity Level Scale (SGPALS) within 48 h from admission^22^. Occupational therapists screened and registered cognitive function using the MoCA^23^ during the stay at the stroke units where the median length of stay was 12–15 days 2014–2015, and 7–10 days 2016–2018^24^. All the physiotherapists and occupational therapists at the stroke units were trained to make assessments according to local guidelines. Cognitive performance in different daily activities was also routinely assessed by occupational therapists if appropriate.

### Variables

Descriptive variables were patient characteristics, stroke attributes and complications, length of stay at the stroke unit, and discharge destination. The dependent variable was cognition screened with the MoCA. The MoCA is a brief cognitive screening tool with high sensitivity and specificity for detecting mild cognitive impairment^23^ and is feasible in acute and subacute^25^ stroke. The score ranges from 0 to 30, where a score of ≤ 25 indicates cognitive impairment and ≥ 26 points indicates intact cognition; MoCA scores were thus dichotomized in the analyses^23^. No information about pre-stroke cognitive status was registered, although variables describing independent living in the community were registered (including the variables living in one’s own home, having no need of help, and independence in walking, transfers, eating, dressing, bathing, and toilet visits). Independence in all-day living is associated with intact cognition^26^, therefore previously dependent patients were excluded from the study as an assumption of normal cognitive performance before stroke. Furthermore, variables concerning prerequisites to use the MoCA were included to compare the study sample with the excluded patients. The variables were communication difficulties, visual or arm impairment from the NIHSS items.

The independent variables for the regression model building were selected as cardiovascular risk factors and variables previously associated with impaired cognition^4,7^. Included variables were demographic factors (age, sex, education), and risk factors for stroke (pre-stroke physical activity, diabetes mellitus, smoking, atrial fibrillation, smoking, and hypertension treatment or statin treatment) in line with previous studies^4,7^ with the additional variables previous TIA, reperfusion therapies, and stroke severity. Pre-stroke physical activity was categorized into four levels according to the SGPALS^27,28^:

1. Physically inactive: being almost completely inactive: reading, watching television, watching movies, using computers, or doing other sedentary activities during leisure time.
2. Some light physical activity (light physical activity): being physically active for at least 4 h/week, for example, riding a bicycle or walking to work, walking with the family, gardening, fishing, table tennis, bowling, etc.
3. Regular physical activity and training (moderate physical activity): spending time on heavy gardening, running, swimming, playing tennis, badminton, calisthenics, and similar activities for at least 2–3 h/week.
4. Regular hard physical training for competition sports (high physical activity): spending time in running, orienteering, skiing, swimming, soccer, European handball, etc., several times/week.

The physiotherapist assessed pre-stroke physical activity levels by asking the patient: “How physically active were you before your stroke? Try to estimate an average over the past year.” The patients were asked to be as specific as possible regarding frequency, intensity, duration, and type of physical activity, with information often confirmed by next of kin. For descriptive purposes, the SGPALS data were divided into three groups, with levels 3 and 4 merged due to there being very few patients (n = 4) at level 4, both in descriptive analyses as well as in the regression analysis. Therefore SGPALS 3–4 is called moderate physical activity hereafter. Stroke severity at hospital admission was assessed using the National Institutes of Health Stroke Scale (NIHSS score 0–42)^29^, and was divided into mild stroke (0–5) and moderate stroke (6–14)^21^. Age was a continuous variable, while other variables were dichotomized as follows: sex as men or women, education as ≤ 12 or > 12 years, and the remaining variables as yes or no.

### Data analyses

For descriptive purposes, continuous variables are presented as means and standard deviations (SD) and categorical variables as numbers (n) and percentages. Drop-out analyses were made with χ^2^ tests for sex, NIHSS items (communication deficits, visual impairment, and arm paresis) related to patients´ ability to be screened with the MoCA, and the Mann–Whitney U test for age and stroke severity (as the NIHSS is an ordinal scale). In the drop-out group, reasons for forgoing MoCA assessment were registered and are presented as numbers. Further differences between groups for patients with cognitive impairment (MoCA ≤ 25), and intact cognition (MoCA ≥ 26 points) were tested using χ^2^ tests for categorical variables and the Mann–Whitney *U* test for continuous variables. Statistical analyses were performed using IBM SPSS Statistics 27. The significance level was set to α < 0.05.

Regression model building commenced with the selection of the independent variables. The next steps were to check the assumptions, with cross tabulation between the dichotomized MoCA and all independent variables to find redundant groups (defined as ≤ 10 events per variable)^30^, followed by rank correlation analyses between the continuous and ordinal variables. Thereafter, univariable binary logistic regression analyses were performed to investigate if the independent variables influenced the dependent variable (intact cognition screened with the MoCA). Subsequently, variables with *P* < 0.25^31^ were included in a multivariable binary logistic regression analysis (backward) resulting in a final model with the remaining variables to predict intact cognition after stroke. The results of the regression analyses are presented as odds ratios (OR) and 95% confidence intervals (95% CI). Furthermore, the regression model (set of predictor variables) was checked with the Omnibus test (a value of < 0.05 is in support of the model) and the Hosmer and Lemeshow goodness-of-fit test (a value of > 0.05 supports the model). For exploring the variability of the dependent variables explained by the regression model the Cox-and-Snell and Nagelkerke R^2^ values were evaluated. Area under the curve (AUC) was calculated in a receiver operating characteristic curve. The AUC value should exceed 0.5, and the closer to 1 the better fit of the model. The stability of the model was checked with five-fold cross-validation with repeated random subsampling, where the variables in a randomly selected 80% of the study sample were compared with the variables in the final model from the total study sample. This involved repeating the multivariable binary logistic regression analyses for the subsamples five times, resulting in five models for comparison. Additionally, on variable level the OR (95% CI) were compared, and on model level the AUC values were compared for all models.

### Licenses

The data were analysed using IBM SPSS Statistics for Windows (Version 26.0. Armonk, NY, IBM Corp. Released 2019). The figures were created using the GraphPad prism (Version 9, GraphPad Software, Inc., http://www.graphpad.com). The licences for SPSS and GraphPad prism were provided by the University of Gothenburg. A permission request was sought and accepted the use of the MoCA^©^ test without changes or adaptations.

## Results

### Descriptive characteristics of the study sample

**Figure 1:**
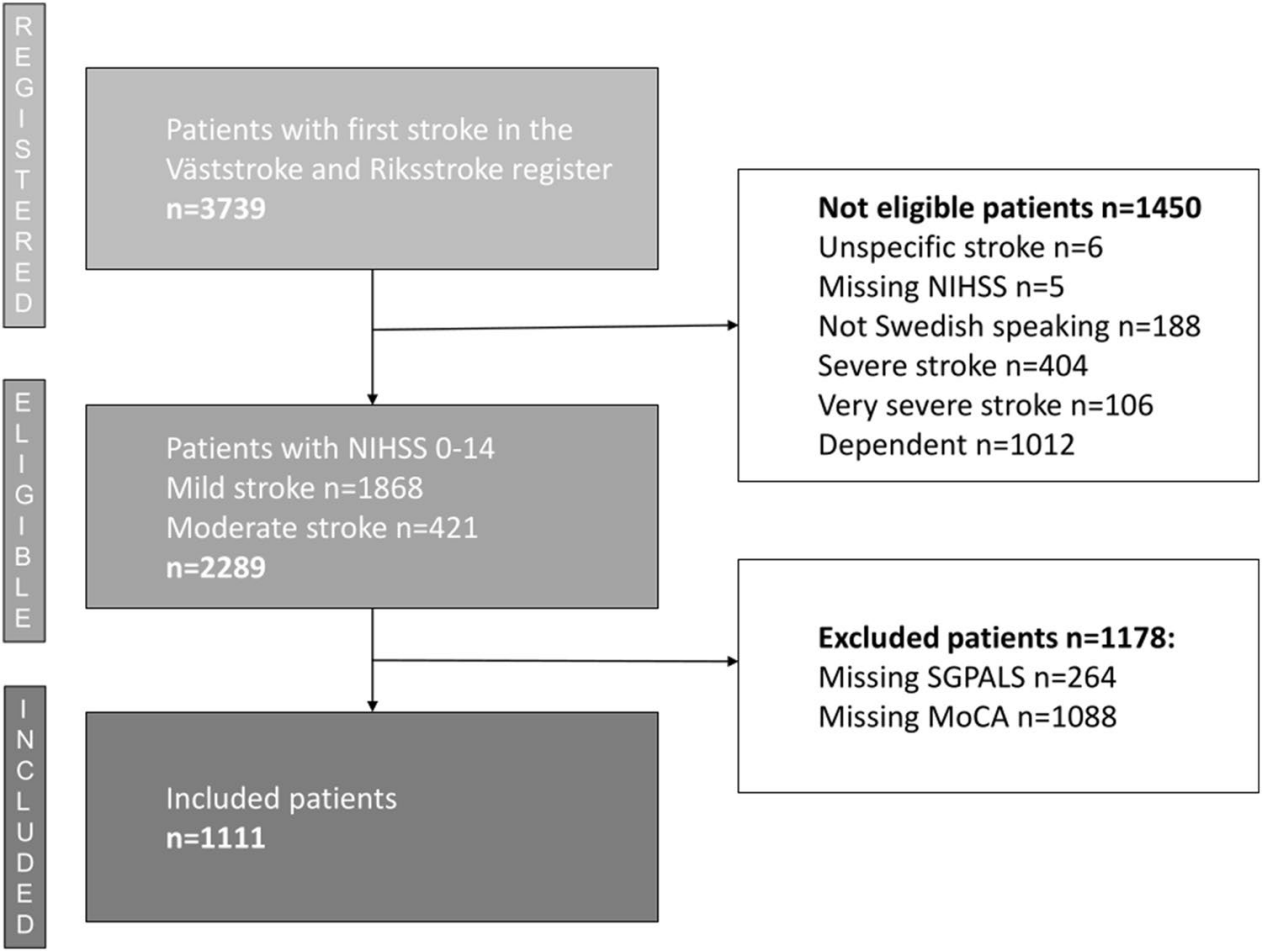
Flow chart of the study participants. Physical activity level was assessed using the Saltin-Grimby Physical Activity Level Scale (SGPALS), and cognition was screened using the Montreal Cognitive Assessment (MoCA).

**Figure 2:**
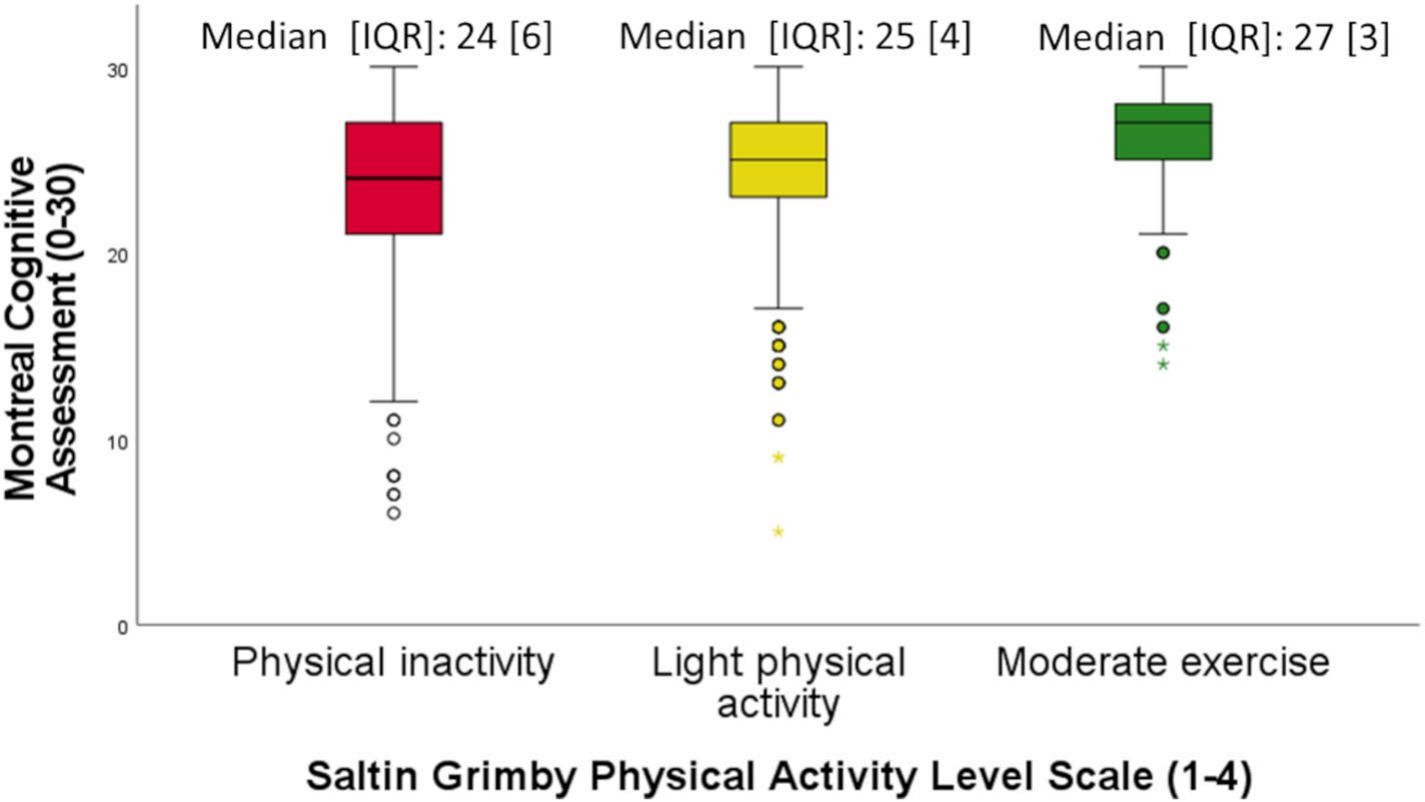
Box plots of patients with different levels of pre-stroke physical activity. Physical activity level was assessed using the Saltin-Grimby Physical Activity Level Scale, (SGPALS levels 1–4) on post-stroke cognition (screened with Montreal Cognitive Assessment, MoCA score 0–30) where ≤ 25 points indicate cognitive impairment, and ≥ 26 points indicate intact cognition. Median and interquartile range (IQR) for MoCA are presented for the separate SGPALS levels.

The study sample consisted of 1111 patients with a first stroke; see the flow chart in Fig. 1. The mean patient age was 69.8 years, 39.8% were females, 93.4% had an ischemic stroke, the median NIHSS score was 1, 61.5% were pre-stroke physically active, 53.1% had a possible cognitive impairment when screened with the MoCA, and the median length of inpatient stay at the stroke unit was 6 days (range 0–61 days) with a mean of 9.1 days, see Table 1. Patients with different levels of pre-stroke physical activity and their post-stroke cognition are visualized in Fig. 2.

**Table 1:** Descriptive characteristics of the study sample, presented in groups based on cognitive function, (cognitive impairment MoCA 1–25, and intact cognition MoCA 26–30) screened using the Montreal Cognitive Assessment (MoCA).

Characteristics	MoCA 1–25 (n = 590, 53.1%)	MoCA 26–30 (n = 521, 46.9%)	Total study sample (n = 1111)	Excluded patients (n = 1178)
	*Mean (SD)*	*Mean (SD)*	*Mean (SD)*	*Mean (SD)*
Age, years	72.8 (12.4)	66.5 (13.4)	69.8 (13.3)	70.1 (14.1)
Length of stay at stroke unit, d				
	10.5 (8.4)	7.4 (6.5)	9.1 (7.7)	11.8 (12.9)
	*n (%)*	*n (%)*	*n (%)*	
Sex, women	249 (42.2)	193 (37.0)	442 (39.8)	524 (44.5)
Education ≤ 12 years	311 (52.7)	215 (41.3)	526 (47.3)	44 (4.6)
**Conditions prior to stroke:**				
Diabetes, yes	109 (18.5)	61 (11.7)	170 (15.3)	199 (16.9)
Smoking, yes	95 (16.1)	70 (13.4)	165 (14.9)	155 (13.2)
Statin treatment, yes	136 (23.1)	90 (17.3)	226 (20.3)	253 (21.5)
Hypertension treatment, yes	340 (57.6)	255 (48.9)	595 (53.6)	642 (54.5)
Previous TIA, yes	36 (6.1)	26 (5.0)	62 (5.6)	71 (6.0)
Atrial fibrillation, yes	123 (20.8)	76 (14.6)	199 (17.9)	241 (20.5)
**Pre-stroke PA level (SGPALS):**				
1. Physically inactive	257 (43.6)	171 (32.8)	428 (38.5)	423 (35.9)^1^
2. Light PA	298 (50.5)	275 (52.8)	573 (51.6)	410 (44.9)^1^
3–4. Moderate PA	35 (5.9)	75 (14.4)	110 (9.9)	81 (8.9)^1^
**Stroke attributes:**				
Cerebral hemorrhage	50 (8.5)	23 (4.4)	73 (6.6)	136 (11.5)
Ischemic stroke	540 (91.5)	498 (95.6)	1038 (93.4)	1042 (88.5)
Thrombolysis, yes	68 (11.5)	59 (11.3)	127 (11.4)	126 (10.7)
Thrombectomy, yes	27 (4.6)	17 (3.3)	44 (4.0)	68 (5.8)
**Stroke severity:**				
NIHSS, median [IQR]	2 [4]	1 [2]	1 [3]	2 [6]
Mild Stroke	499 (84.6)	487 (93.5)	986 (88.7)	882 (74.9)
Moderate stroke	91 (15.4)	34 (6.5)	125 (11.3)	296 (25.1)
**Cognition after stroke:**				
MoCA median [IQR]	22 [5]	27 [3]	25 [5]	25 [5]^1^
Assessed in daily activities	349 (61.7)	123 (24.3)	472 (44.0)	375 (45.0)^1^
**After discharge from hospital admitted to:**				
Own home no help	381 (64.6)	449 (86.2)	830 (74.7)	699 (59.3)
Own home with help	133 (22.5)	34 (6.5)	167 (15.0)	114 (9.7)
Nursing home	21 (3.6)	8 (1.5)	29 (2.6)	146 (12.4)
Hospital/rehab ward	47 (8.0)	27 (5.2)	74 (6.7)	154 (13.1)
**Complications during hospital care:**				
New stroke	4 (0.7)	3 (0.6)	7 (0.6)	41 (3.6)
Myocardial infarction	2 (0.3)	3 (0.6)	5 (0.5)	12 (1.0)
Deceased	0	1 (0.2)	1 (0.1)	61 (5.2)

Percent (%) as valid percent. N indicates numbers; SD, standard deviation; IQR, interquartile range; d, days; PA, physical activity; TIA, transient ischemic attack; NIHSS, National Institutes of Health Stroke Scale with mild stroke (0–5), and moderate stroke (6–14); SGPALS, Saltin-Grimby Physical Activity Level Scale. Missing in study sample (n = 1111): length of hospital stay at the stroke unit (n = 61), diabetes (n = 1), smoking (n = 82), statin treatment (n = 2), hypertension treatment (n = 1), previous TIA (n = 5), new atrial fibrillation (n = 1), thrombolysis (n = 4), thrombectomy (n = 45), assessment in daily activities not appropriate (n = 305), after discharge from hospital admitted to (6), new stroke (n = 12), myocardial infarction (n = 7). ^1^ Indication of more than 20% internal missing values in the group of excluded patients.

Among the not eligible patients (n = 1450) the patients with severe or very severe stroke (n = 510) and the previously dependent patients (n = 1012) had a median NIHSS score of 19, and 6 respectively. The patients were cognitively assessed in different activities 54% and 45%, respectively. Additionally, 8.0% and 17.5% of the not eligible patients were screened with the MoCA, respectively with a median MoCA score of 21 points in both groups.

### Analyses between groups of patients, study sample versus excluded patients

When comparing the final study sample (n = 1111) with the group of excluded patients (n = 1178) the excluded group included more women (*P* < 0.023) and had more severe strokes (*P* < 0.001) with the NIHSS median score of 2, as seen in Table 1. Of the eligible patients (n = 2289) 52.5% were screened with the MoCA, and 39.4% were not screened due to registered reasons. In the excluded group, only 90 of 1178 patients were screened with MoCA. Reasons for forgoing MoCA assessment were, for example, aphasia (n = 101), dementia/severe cognitive decline/delirium (n = 73), and visual impairment (n = 47). Patients in the excluded group had properties on NIHSS items hindering MoCA assessment to a greater extent than those in the study group. They more often had arm impairment (right and/or left) (20.2%, *P* = 0.001), communication difficulties (dysarthria and/or aphasia) (36.0%, *P* < 0.001), and/or visual impairment (visual fields and/ocular movements) (18.5%, *P* = 0.004) compared with the study group (14.5%, 28.0%, and 13.5%, respectively).

### Analyses between groups of patients, with possible impaired cognition versus intact cognition

In analyses between the group with impaired cognition (MoCA ≤ 25) and intact cognition (MoCA ≥ 26), the group with lower MoCA values were significantly older (*P* < 0.001, had increased length of hospital stay at the stroke unit (*P* < 0.001), more often cerebral hemorrhage, less often ischemic stroke (*P* = 0.006), more moderate than mild stroke severity (*P* < 0.001), more presence of diabetes (*P* = 0.002), more hypertension (*P* = 0.004) and statin (*P* = 0.017) treatment, a lower level of pre-stroke physical activity (*P* < 0.001), shorter education (*P* = 0.007), and were more often assessed in daily activities (*P* < 0.001).

### Associations between the independent variables and intact cognition

**Figure 3:**
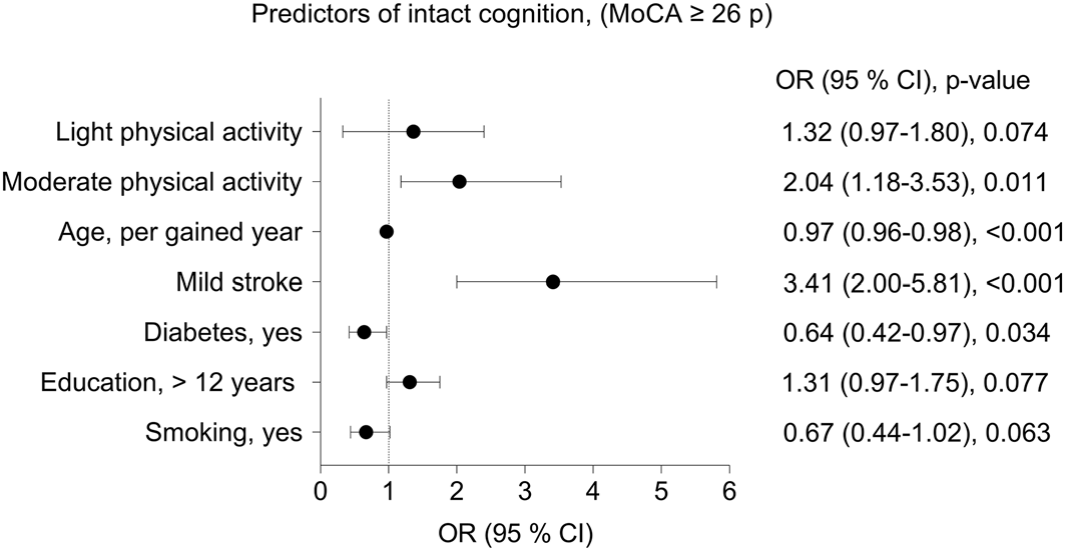
Associations of prognostic factors for intact cognition presented with Forest plots. Associations are presented as values for odds ratio (OR) and 95% confidence interval (95% CI). Intact cognition was defined as a score of ≥ 26 points on the Montreal Cognitive Assessment (MoCA 0–30), physical activity on the Saltin-Grimby Physical Activity 4-Level Scale (SGPALS), and stroke severity according to the National Institutes of Health Stroke Scale (NIHSS 0–42) as mild (0–5 points) or moderate (6–14 points).

The independent variables (pre-stroke physical activity, age, sex, diabetes, atrial fibrillation, statin treatment, hypertension treatment, stroke severity, smoking and education) had *P* < 0.25 in the univariable binary logistic regression (Supplemental Table S1) and were thereafter included in the multivariable binary logistic regression analysis (Supplemental Table S2). This resulted in a final model where pre-stroke physical activity was associated with intact cognition: OR (95% CI) 1.32 (0.97- 1.80) for light physical activity, and 2.04 (1.18-3.53) for moderate physical activity. In addition to physical activity, the final model also associated younger age, being non-diabetic, non-smoking, higher level of education, and less severe stroke (more mild than moderate) with higher odds for intact cognition, see Fig. 3. The explanatory value of the final model varied between 11.2% (Cox and Snell R square) and 14.9% (Nagelkerke R square). Tests in support of the model were the Hosmer and Lemeshow test with a value of 0.962, and the Omnibus test *p* value < 0.001 and AUC was 0.696. The stability of the final model was confirmed by similar variables and AUC values when compared with the fivefold cross-validations presented in Supplemental Table S2.

## Discussion

Physical activity before stroke is associated with intact cognition in patients with mild and moderate stroke assessed during the acute and subacute phases after stroke. Moderate physical activity is more beneficial compared to light. Furthermore, in addition to being physically active, patients with younger age, non-diabetic, non-smoking, a higher level of education, and less severe stroke (more mild than moderate) have higher odds for intact cognition.

The associations of pre-stroke physical activity on cognitive performance reported in this study is confirmed by a study where faster processing speed was reported (after 1 and 6 months) for people who were physically active before stroke^16^. However, no association was found for global cognition screened with the Mini-mental state examination (MMSE) after 6 months^16^. Another study reported no association with cognitive decline for a combination of pre-stroke physical activity and smoking^7^. Related to this topic a study investigated the influence of several different activities (intellectual, recreational, social, or physical activities) before stroke on post-stroke dementia (assessed with the MMSE) and found that more activities were associated with a lower risk of post-stroke dementia^17^. Moreover, regular participation in intellectual activities and walking were associated with a reduced short-term risk of post-stroke dementia, while physical exercise was not^17^. Comparisons between the present study and previous studies are limited by the different assessments used for physical activity and cognition, and different timing. When screening for mild cognitive impairment, the MoCA has high sensitivity and specificity^23^, compared with the MMSE which is not sensitive enough to assess cognition when several cognitive domains are affected^6^. In the present study, MoCA assessment was performed on average within 9 days. Cognitive functions assessed early after stroke with the MoCA are associated with early dependency in activities of daily living^8^ and functional disability after 3 months^26^.

A strength of the study is the use of the MoCA, since it is valid, clinically feasible, and can detect a range of cognitive impairments after stroke^32^. The MoCA is suited to detect mild cognitive impairment after stroke^23^. While it would have been of interest to study cognition among patients with more severe stroke, this was not feasible using the MoCA: it demands holding a pencil, vision, and language, and therefore patients with severe stroke were excluded from this study. The mentioned limitation of the MoCA can partly be compensated for with MoCA versions of visual impairment and use of hearing aids. Cognitive screening early after stroke is recommended internationally^33,34^, and can help in rehabilitation goal setting and discharge planning^35^. The cognitive screenings can also be followed by more comprehensive cognitive assessments^35^ in the late subacute or chronic phases after stroke^25^. However, a majority of studies are conducted 6 months post-stroke therefore more research conducted early after a stroke is needed^36^. Another strength is that this was a register-based study from a consecutively collected large data sample, in a context of tax-supported public healthcare and high register coverage. The results from this present study can be generalized to similar settings for people who are previously independent or have a mild or moderate stroke. One limitation is the lack of pre-stroke cognitive status or dementia. To ensure pre-stroke cognitive performance, previously dependency was an exclusion criterion since independence in all-day living is associated with intact cognition^26^. There is a risk of selection bias due to the decision to only include previously independent patients with mild or moderate stroke in this study. However, few patients with severe stroke or previous dependency were screened with the MoCA in the data base, and instead often cognitively assessed in daily activities. Furthermore, the MoCA is a generic assessment and a stroke specific instrument for cognitive screening may cover all stroke severities. Barriers to cognitive screening at a stroke unit are foremost mobility and communication problems^37^. A second limitation is the use of data from registers where variables are predefined, leaving variables of interest such as patients´ pre-stroke cognition, unaddressed. Quality registers are often missing internal values in individual variables. A third limitation is that the data collected in a clinical setting can result in reporting bias, due to the number of health-care staff involved in data collection. Additionally, there is a risk of recall bias in the self-reported physical activity level scale. To reduce this bias, assessment was performed by the physiotherapist together with follow-up questions and confirmation from next of kin when possible. Another limitation of the SGPALS is its lack of agreement with current international recommendations about physical activity^38^.

This study elucidates the benefits of physical activity and provides additional knowledge regarding associations with cognition, often affected after stroke. Cognitive deficits after stroke can be debilitating and difficult to understand for both patients and people in their environment^39^. Furthermore, the results support the importance of physical activity in promoting public health and stroke prevention. Healthcare professionals play an important role in supporting physically inactive individuals in making lifestyle changes, with interventions including exercise recommendations, prescriptions for physical activity, and structured follow-ups. Future research should further explore associations of pre-stroke physical activity and its long-term consequences after stroke. Moreover, both volume and location are of interest when performing research on stroke outcomes. Few previous studies have explored the association between pre-stroke physical activity and radiologic findings in humans^13,40^. Additionally, objective assessment tools with consistency between studies are needed for comparisons between studies. The different aspects of physical activity, such as the intensity, frequency, duration, and type of exercise, also need further exploration. Longitudinal and prospective epidemiological studies can also contribute to knowledge about physical activity.

In conclusion, pre-stroke physical activity is associated with intact cognition early after stroke in previously independent patients with mild and moderate stroke. Moderate physical activity, such as exercise at hours 2–3 times/week, seem to be more beneficial compared to light physical activity, such as walking at least 4 h/week. In addition to pre-stroke physical activity, people with younger age, being non-diabetic, non-smoking, a higher level of education, and less severe stroke (more mild than moderate) have higher odds for intact cognition after stroke.

## Supplementary Information

Supplementary Information 1.

Supplementary Information 2.

Supplementary Information 3.

## Data Availability

The trial is registered at www.researchweb.org, project number 214641. According to Swedish regulations (http://www.epn.se/en/start/regulations/), the permission to use data requires application to and approval from the ethics board. Thus, the complete dataset cannot be made publicly available for ethical and legal reasons. Data may be available to researchers upon request by email to the principal investigator ks.sunnerhagen@neuro.gu.se.
