# Supplementary material for: A register-based study on associations between pre-stroke physical activity and cognition early after stroke (part of PAPSIGOT)
Source: Sci Rep. 2022 Apr 6;12:5779. doi: 10.1038/s41598-022-09520-2 (PMC8986803; doi:10.1038/s41598-022-09520-2)
Supplement: Supplementary file 2 — Supplementary Information 2. [file 41598_2022_9520_MOESM2_ESM.pdf]

Table S1. The results of univariable binary logistic regression analyses for predicting intact cognition (Montreal Cognitive Assessment, MoCA ≥ 26 points), n=1111.

Independent variables	Reference category	Cox and Snell square−Nagelkerke R square	Unstandardized coefficients B (Std Error)	OR (95% CI)	p-value
**Pre-stroke PA SGPALS 2**	Physical inactivity SGPALS 1	0.026−0.034	0.33 (0.13)	1.39 (1.08-1.79)	**0.011**
**Pre-stroke PA SGPALS 3-4**			1.17 (0.23)	3.22 (2.06-5.03)	**<0.001**
**Age**	Continuous	0.057−0.076	-0.04 (0.01)	0.96 (0.95-0.97)	**<0.001**
**Sex (female)**	Male	0.003−0.004	-0.22 (0.12)	0.81 (0.63-1.03)	**0.080**
**Smoking**	No	0.002−0.003	-0.26 (0.17)	0.77 (0.55-1.08)	**0.124**
**Diabetes**	No	0.009−0.012	-0.54 (0.17)	0.58 (0.42-0.82)	**0.002**
**Previous TIA**	No	0.001−0.001	-0.21 (0.27)	0.81 (0.48-1.36)	0.427
**Atrial fibrillation**	No	0.007−0.009	-0.43 (0.16)	0.65 (0.47-0.89)	**0.007**
**Statin treatment**	No	0.005−0.007	-0.36 (0.15)	0.70 (0.52-0.94)	**0.017**
**Hypertension treatment**	No	0.007−0.010	-0.35 (0.12)	0.71 (0.56-0.90)	**0.004**
**Reperfusion therapies**	No	0.000−0.000	-0.064 (0.18)	0.94 (0.66-1.33)	0.722
**Education >12 years**	≤12 years	0.013−0.017	0.46 (0.12)	1.59 (1.25-2.01)	**<0.001**
**Mild stroke severity**	Moderate stroke	0.020−0.027	0.96 (0.21)	2.61 (1.73-3.95)	**<0.001**

PA=physical activity. SGPALS=Saltin Grimby Physical Activity 4 Level Scale. Mild stroke (0-5 points on the NIH Stroke Scale, NIHSS) Moderate stroke (6-14 on the NIHSS). OR=Odds Ratio. CI=Confidence Interval. Significant p-values in bold text were included in the multivariable binary logistic regression analysis.
