# Supplementary material for: A register-based study on associations between pre-stroke physical activity and cognition early after stroke (part of PAPSIGOT)
Source: Sci Rep. 2022 Apr 6;12:5779. doi: 10.1038/s41598-022-09520-2 (PMC8986803; doi:10.1038/s41598-022-09520-2)
Supplement: Supplementary file 3 — Supplementary Information 3. [file 41598_2022_9520_MOESM3_ESM.pdf]

Table S2. Results from the stability check of the regression model with five-fold cross-validation. The results from a randomly selected 80% of the study sample (repeated 5 times) were compared with the 100% study sample for variables associated with intact cognition (≥ 26 points with MoCA).

Final model and cross validation models 1-5		Full final model n=1111	Cross validation fold 1 n=901	Cross validation fold 2 n=897	Cross validation fold 3 n=865	Cross validation fold 4 n=915	Cross validation fold 5 n=884
Explanatory variables pooled in the multivariable binary logistic regression models	SGPALS 2, OR (95% CI), p-value	1.32 (0.97-1.80), 0.074	1.49 (1.06-2.09), 0.022	1.34 (0.95-1.89), 0.025	1.33 (0.94-1.89), 0.107	1.48 (1.06-2.07), 0.022	1.34 (0.95-1.88), 0.093
	SGPALS 3-4, OR (95% CI), p-value	2.04 (1.18-3.53), 0.011	2.57 (1.40-4.71), 0.002	2.24 (1.22-4.13), 0.009	2.06 (1.10-3.84), 0.024	2.73 (1.49-5.00), 0.001	2.27 (1.25-4.12), 0.007
	Age, per year, OR (95% CI), p-value	0.97 (0.96-0.98), <0.001	0.97 (0.96-0.98), <0.001	0.96 (0.95-0.98), <0.001	0.97 (0.96-0.98), <0.001	0.97 (0.95-0.98), <0.001	0.97 (0.96-0.98), <0.001
	Diabetes, yes, OR (95% CI), p-value	0.64 (0.42-0.97), 0.034	0.62 (0.39-0.97), 0.037	0.57 (0.36-0.90), 0.017	0.51 (0.32-0.81), 0.005	0.59 (0.37-0.94), 0.027	
	NIHSS 0-5, OR (95% CI), p-value	3.41 (2.00-5.81), <0.001	2.75 (1.56-4.85), <0.001	3.43 (1.90-6.22), <0.001	3.76 (2.07-6.83), <0.001	3.71 (2.06-6.67), <0.001	3.01 (1.65-5.48), <0.001
	Education >12 years, OR (95% CI), p-value	1.31 (0.97-1.75), 0.077	1.39 (1.00-1.93), 0.048		1.45 (1.03-2.02), 0.031		1.41 (1.02-1.95), 0.039
	Smoking, yes, OR (95% CI), p-value	0.67 (0.44-1.02), 0.063		0.59 (0.37-0.94), 0.025			
	Hypertension, yes, OR (95% CI), p-value			1.36 (0.96-1.92), 0.089			
Evaluation of the cross validated multivariable binary logistic regression models	Cox and Snell square	0.112	0.113	0.116	0.120	0.121	0.094
	Nagelkerke R square	0.149	0.150	0.155	0.160	0.161	0.125
	Hosmer and Lemeshow test	0.962	0.447	0.038	0.669	0.673	0.651
	Area Under the ROC curve	0.696	0.697	0.704	0.697	0.703	0.681
	Omnibus test p-value	<0.001	<0.001	<0.001	<0.001	<0.001	<0.001

MoCA= Montreal Cognitive Assessment. SGPALS=Saltin Grimby physical activity scale, SGPALS 2=light physical activity, SGPALS 3-4=moderate physical activity. OR=Odds Ratio. CI=Confidence Interval. NIHSS=National Institutes of Health Stroke Scale. ROC=Receiver Operating Characteristic.
